# Cd(II) Adsorption on Different Modified Rice Straws under FTIR Spectroscopy as Influenced by Initial pH, Cd(II) Concentration, and Ionic Strength

**DOI:** 10.3390/ijerph16214129

**Published:** 2019-10-26

**Authors:** Shuai Wang, Wanhong Li, Xinhua Yin, Nan Wang, Shuai Yuan, Ting Yan, Shuang Qu, Xiangbo Yang, Dianyuan Chen

**Affiliations:** 1 College of Agriculture, Jilin Agricultural Science and Technology University, Jilin 132101, China; wangshuai419@126.com (S.W.); liwanhong123@126.com (W.L.); wangnan664806@126.com (N.W.); ys1178968997@163.com (S.Y.); yannting@126.com (T.Y.); qushuang0602@163.com (S.Q.); 2 Department of Plant Sciences, The University of Tennessee, Knoxville, TN 37996, USA

**Keywords:** rice straw, Cd(II), FTIR, adsorption, influencing factor

## Abstract

Rice straw is a kind of low-cost biosorbent. Through mechanical crushing, pyrolysis, incineration, and citric acid (CA) modification, it could be converted to rice straw powder (Sp), biochar (Sb), ash (Sa), and modified rice straw (Ms) accordingly. Using rice straw as an adsorbent, the influence of pH value (2, 4, and 9), initial Cd(II) concentration (0, 200, and 800 mg/L), and ionic strength (0, 0.2, to 0.6 mg/L) on the adsorption capacity for Cd(II) were examined with three replicates, and the relevant mechanisms were explored using Fourier transform infrared (FTIR) technology. Results showed that the modifications could improve the adsorption capacity of Cd(II) by changing their chemical structures. The products (Sb and Sa) of the pyrolysis and incineration of rice straw contained fewer hydroxyl and alkyl groups, but more Si–O groups. Citric acid modification removed a portion of silica in rice straw, increased its carboxylic content, and made more –OH groups exposed. Compared with Sp, Sb, Sa, and Ms were more likely to act as *π* donors in the Cd(II) sorption process and exhibited more carboxyl binding. The bands of C = C, –O–CH_3_, and the O–H, carboxyl, Si–O–Si or Si–O groups were involved in the Cd(II) sorption process. The adsorption amount of Cd(II) by the four adsorbents increased with the increase in the pH value of the solution and the initial Cd(II) concentration. Affected by pH in a solution, ion exchange, surface complexation, and precipitation were the major adsorption mechanisms. Further, under the influence of the initial Cd(II) concentration, electrostatic attraction played a leading role. With no interference by ionic strength, all the adsorbents had the greatest adsorption amount of Cd(II), and the intensity of O–H vibration was also the weakest; ion exchange was the most important mechanism in this process. Regardless of the influencing factors, Sa, with the greatest specific surface area, had an absolute advantage in the adsorption capacity of Cd(II) over Sp, Sb, and Ms.

## 1. Background

Cadmium (Cd) is one of the most toxic heavy metal elements. Cd(II) has become a significant concern owing to its high mobility and biological accumulation. Cui et al. [1] and Peng et al. [2] reported that Cd(II) is ubiquitous in the world and can cause kidney and bone damage after long-term exposure. Conventional treatment approaches for removing Cd(II) ions from aqueous systems include coagulation, ion exchange, membrane separation, adsorption and chemical precipitation. However, Song et al. [3] found that adsorption is a better choice than the others because of its lower cost. 

Rice (*Oryza sativa* L.) is the second-largest cereal crop in the world. It is used as a staple food by over half of the world’s population [4]. Approximately 100 million tons of rice straw are produced in China each year [5]. However, Chinese farmers burn large amounts of rice straw after the harvest season, resulting in severe air pollution problems such as haze [6]. As a lignocellulosic waste, rice straw mainly consists of cellulose, hemicellulose, and lignin [4,7]. Even though the adsorption capacity of crude rice straw is generally low [8], its richness in hydroxyl groups (−OH) provides a lot of reactive sites for surface modification to be converted into a more desired adsorbent [9]. The surface modification of adsorbents to maximize their physicochemical and adsorption properties is critical for the development of new technologies for environmental remediation [10]. For instance, rice straw pyrolysis in an oxygen-limited environment can produce biochar with a larger surface area and more hydroxyl groups [11]. Chemical modification of rice straw with citric acid, phosphoric acid, etc., can enhance the number of surface carboxyl groups and increase the cation exchange capacity of the material [12,13]. The functional groups such as carboxyl, silanol, etc., present in rice husk ash are favorable for Cd(II) adsorption [14]. Its porous structure has a larger specific surface area, and this morphological property is conducive to the uptake of metal ions [15]. Hence, the modification has the potential of transforming rice straw into a more valuable adsorbent to remove heavy metal pollution in water [10]. A large number of investigations have reported the adsorption of heavy metals in wastewater by agricultural solid waste and its modified materials. Rocha et al. [16] used waste rice straw as a adsorbent to adsorb Cu(II), Zn(II), Cd(II) and Hg(II) from aqueous solutions and found that the effect of removing Cd(II) by rice straw was the most effective. The modified rice straw is a good candidate for the biodegradable ion exchange resin [13]. The modified rice husk had faster kinetics and higher adsorption capacities than the natural rice husk, and –CO–, –OH, –C–OH, –Si–H, Si–O–Si and –Si–OH from the natural rice husk were effective in the adsorption Cd(II) [17]. The soybean straw was citric acid (CA) modified to introduce high amounts of free carboxyl groups and enhance its adsorption capacity of Cu(II) [8]. Zheng et al. [18] described the adsorption of Cd(II) from aqueous solution using acrylonitrile-modified corn stalk. Barley straw was thermochemically modified with CA to improve the sorption capacity of Cu(II) from aqueous solutions [19]. Sharma et al. [4] evaluated the efficiency of rice straw bed columns for removing and recovering Ni(II) from contaminated industrial effluent through a continuous system. Cheng et al. [20] investigated the adsorption of pyrolyzed biochars prepared from 22 kinds of feedstocks for Cd(II) in aqueous solutions and pointed out that peanut husk biochar could be used as a good remediating material for the removal of Cd(II) from contaminated environmental matrixes.

Regarding the research on the adsorption mechanism, Cu(II) and Zn(II) adsorption by rice straw biochar were closely related to the exchangeable cation [21]. The carboxyl and hydroxyl functional groups were mainly responsible for the sorption of Cd(II) on mango peel waste [22]. The ether, alcoholic and amino groups from the chestnut shell were involved in the adsorption process for Cd(II) [23]. The sorption of Cd(II) on mungbean husk was found to be dependent on pH and the initial concentration of Cd(II) in solution, and Fourier transform infrared (FTIR) spectra showed that the principal functional groups from the mungbean husk that participated in the sorption process included carboxyl, hydroxyl, and amino groups [24]. The possible mechanisms for Cd(II) sorption on adsorbent included [25,26]: (i) Cd(II) precipitate with minerals (e.g., PO_4_^3−^, CO_3_^2−^, and SiO_3_^2−^); (ii) metal ion exchange with Cd(II) (such as K^+^, Ca^2+^, Na^+^, Mg^2+^); (iii) surface complexation between Cd(II) ion and O-containing functional groups (e.g., –OH, –R–OH, –COOH); and (iv) coordination of Cd(II) ion with *π* electrons in unsaturated bonds (e.g., –CH, C = C, C = O) [25,27]. 

However, for the same adsorbent, it was treated with different modification methods, and then the research in the adsorption mechanism for a certain heavy metal was relatively rare. In this research, the rice straw was modified by four different methods. Under the different pH values, initial Cd(II) concentrations, and ionic strengths, the adsorption capacity and mechanism of the four adsorbents to Cd(II) were explored by atomic absorption spectrophotometer (AAS) and Fourier transform infrared (FTIR) techniques. The objectives were to: (1) determine the adsorption capacity of Cd(II) on the four adsorbents in aqueous solution under the different influencing factors, and (2) understand the mechanisms of Cd(II) removal from water by the four adsorbents on a qualitative and quantitative basis.

## 2. Materials and Methods

### 2.1. Materials

Rice straw was collected from the experimental station at Jilin Agricultural Science and Technology University, Jilin, China. The rice straw was washed after harvest with tap water and then rinsed with distilled water and air dried for 2 weeks; rice straw powder (Sp): the air-dried straw was chopped (10 mm), pulverized with a grinder, and passed through a 0.25 mm sieve; rice straw ash (Sa): the rice straw powder was placed in a thin stainless steel tray, and the bottom of the tray was burned with an alcohol lamp while continuously turning over the straw until fully burned; rice straw biochar (Sb): the rice straw powder was placed in a 700 mL ceramic crucible, covered with a lid, and pyrolyzed in the absence of oxygen using a muffle furnace. The temperature was raised at 20 °C/min and kept constant at 500 °C for 2 h [28]. It was cooled at room temperature and ground to pass through a 0.25 mm sieve; modified rice straw (Ms): the rice straw powder was mixed with 0.6 mol/L CA at a ratio of 1:12 (straw/acid, *w*/*v*); after stirring for 30 min at room temperature, the acidic straw slurries were placed in a stainless-steel tray and dried at 50 °C in a forced air oven. After 24 h, the thermochemical reaction between acid and straw was followed by raising the oven temperature to 120 °C for 90 min. After cooling, the reacted products were washed with distilled water to remove excess CA. Lastly, it was dried and cooled in a desiccator until a constant weight was achieved [29].

The N_2_-BET specific surface area (SSA) for Sp, Sa, Sb, and Ms was 1.83, 192.38, 16.47, and 3.17 m^2^/g, respectively, with pore volumes of 0.016, 0.228, 0.045, and 0.020 cm^3^/g, respectively. The high SSA and abundant pore volume of the four adsorbents derived from the rice straw made them promising adsorbents with a good adsorption capacity [3].

### 2.2. Experimental Design

First, 0.05 g of adsorbent was accurately weighed into a 50 mL polyvinyl tube, and then a certain concentration of CdCl_2_ and NaCl (a supporting electrolyte) was added. The total volume of equilibrium solution was 25 mL for all. The adsorption experiments had the following three parts: (1) The initial pH values were set to 2, 4 and 9 with 0.1 mol/L of NaOH or HCl, respectively, the initial Cd(II) concentration was 400 mg/L, and the ionic strength (NaCl) was 0.2 mol/L; (2) The initial Cd(II) concentrations were set to 0, 200 and 800 mg/L, respectively, using 1000 mg/L CdCl_2_, the ionic strength (NaCl) was 0.2 mol/L, and the initial pH value was not adjusted; (3) The ionic strength was set to 0, 0.2 and 0.6 mol/L with 1.0 mol/L NaCl solution, the initial Cd(II) concentration was 400 mg/L, and the initial pH value was not adjusted. There were three replicates of each treatment in each experiment. The adsorption tests were carried out in a constant temperature water bath shaker at 25 °C for a predetermined time, shaking for 10 h and resting for 14 h. Then, the centrifuge tubes were taken out, centrifuged at 12,000 r/min for 10 min, filtered, and diluted to a certain Cd(II) concentration, which was determined by an AAS (TAS 990) produced by Beijing Puxi General Co., Ltd. The amount of Cd(II) adsorption at equilibrium (*q_e_*, mg/g) was calculated using the following equation:

$$q_{e}=(C_{0}-C_{e})V/m_{s}$$

where *C_0_* and *C_e_* (mg/L) were the initial and equilibrium concentration of Cd(II), respectively, *V* (*L*) was the volume of the solution, and *m*_s_ (g) was the mass of the adsorbent. All experiments were conducted with three replicates at room temperature. The Cd(II) concentrations were reported as an average of the three replicates. The precipitated material in the tubes was collected, washed 3 times with deionized water, air dried at 55 °C to a constant mass, and finally stored in a glass desiccator.

### 2.3. FTIR Spectra

The samples stored in a glass desiccator were dried in a vacuum oven at 100 °C for 3 h before measurement. A quantity of 1.5 mg of the sample was compressed under vacuum with 250 mg of KBr at a pressure of 20 MPa. The pellets obtained were analyzed with an FTIR-850 spectrometer from Gangdong Sci and Tech Development Co, Ltd., China, covering a frequency range of 4000 to 400 cm^−1^. Positions and spectral assignments of Cd(II) adsorbed by the four adsorbents derived from rice straw are shown in Table 1.

FTIR spectra were analyzed using the ZWin software supplied with the FTIR-850 spectrometer. The measurement parameters were set as follows: 32 scans with a 4 cm^−1^ resolution, and data interval of 1.93 cm^−1^. The triangle as the apodization mode and the option of collecting background were chosen before collecting samples. The DTGS KBr was set as the detector. The brief steps of spectra processing were described as follows: chose the absorbance as Y-axis format, selected the method of five-point three-time smoothing to smooth the spectra twice, selected the option for automatic baseline correction, marked the absorbance peaks’ position and calculated their area using the peak area tool. Finally, saved the processed spectra with the transmittance (%) as Y-axis as a new CSV file, and plotted them using Origin 8.0 (OriginLab Corp., Northampton, MA, USA).

### 2.4. Data Analysis Method

All calculations and statistical tests were performed using Microsoft Excel 2003 and one-way univariate ANOVA in SPSS 18.0 (IBM Corp., Chicago, IL, USA). All the analyses were considered significant at the *p* < 0.05 level. 

## 3. Results

### 3.1. FTIR Spectra of Four Adsorbents Adsorbing Cd(II) Affected by pH

**Figure 1:**
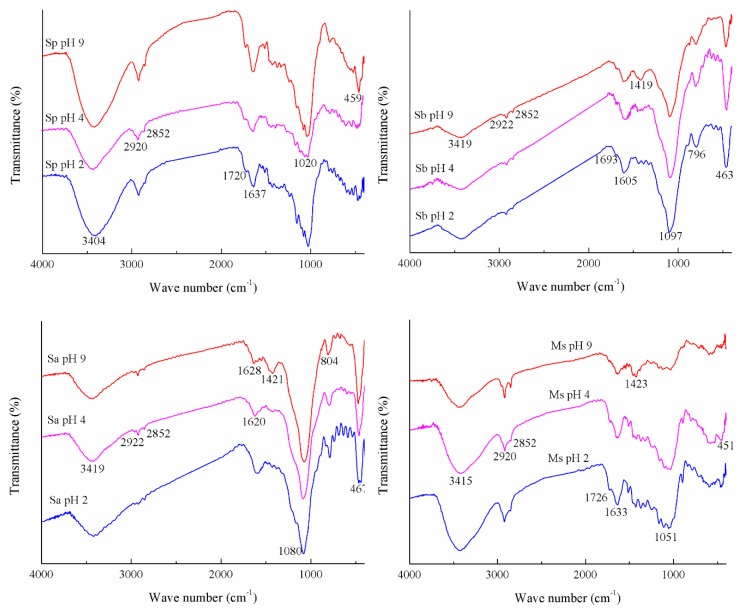
Fourier transform infrared (FTIR) spectra of Cd(II) adsorbed by the four biosorbents under different pH values. Note: the specific experiment conditions are as follows: the initial pH values are set to 2, 4 and 9, respectively, the initial Cd(II) concentration is 400 mg/L, and the ionic strength (NaCl) is 0.2 mol/L. The adsorption of Cd(II) on the rice straw powder (Sp), rice straw ash (Sa), rice straw biochar (Sb), and modified rice straw (Ms) at pH values 2, 4, and 9 are represented by Sp pH 2, 4, 9, Sa pH 2, 4, 9, Sb pH 2, 4, 9, and Ms pH 2, 4, 9, respectively.

As shown in Figure 1 and Table 1, the structures of the four materials after adsorbing Cd(II) had differences and similarities. The similarities were that all four materials had hydroxyl and –CH_2_/–CH_3_ groups, aromatic or benzene rings in lignin, a –O–CH_3_ group from lignin or a Si–O stretching vibration, and a Si–O bending vibration. The differences were that Sp lacked the C–H deformation of the –O–CH_3_ group from lignin and Si–O–Si symmetric stretching, Sa had no C=O group of a carboxylic acid or its ester, and Ms had no Si–O–Si symmetric stretching.

As could be seen from Figure 1 and Table 2, the surface of Sp was mainly composed of some oxygen-containing functional groups (carboxyl, hydroxyl, ether, and ester), exhibiting the potential influence on its adsorption characteristic, which was consistent with the rice husk from the report of Song et al. [3]. Heated rice straw (Sa and Sb) resulted in a significant loss of C–H stretching band (2922 cm^−1^) as compared to Sp, and the weaker intensity of the broad band also occurred at 3419 cm^−1^, which correspond to O–H bond stretching in alcohols and phenols [33]. Different from the above rule, the vibration intensity of the peak at 1080~1097 cm^−1^ of Sa and Sb was increased obviously, and there was a new absorption peak appeared at 796~804 cm^−1^. The C=O bands of carboxyl and ester appeared at the band near 1720 cm^−1^ in Sp, and Ms had the highest intensity at this peak, whereas it disappeared in Sa. This result was similar to that of Park et al. [21]. Compared with Sp, the bands at 1051 and 451 cm^−1^ of Ms attributed to the silica, decreased considerably. An increase in the absorption intensity at 3415 and 2920 cm^−1^ indicated that both the hydroxyl group and the -CH_2_ group increased after CA modification of rice straw. It is worth noting that compared with Sp, the modified adsorbents (Sb, Sa, and Ms) had an increase in the absorption peak intensity at 1605~1633 cm^−1^, and the greatest increase was from Ms, indicating more aromatic structures formed in Sa, Sb, and Ms. Among the four adsorbents, the absorption peaks of 3404~3419 and 1020~1097 cm^−1^ were the two most important peaks. With the increase in pH value, the intensities of the absorption peaks at 3404~3419 and 1605~1637 cm^−1^ decreased gradually after the binding of Cd(II) by the four adsorbents. Except for Sa, the intensity of the absorption peak at 1693~1726 cm^−1^ of the other three adsorbents decreased gradually. Besides, the absorption peaks at 1419~1423 cm^−1^ of Sa, Sb, and Ms gradually appeared. Among the three pH values, the greatest intensity of peak at 1020~1097 cm^−1^ was observed at a pH value of 2 only.

As shown in Table 2, all the amounts of Cd(II) adsorption on the four adsorbents from rice straw increased significantly with the increase in the initial pH values of the solution (2, 4, and 9). Among the four adsorbents, Sa adsorbed the greatest amount of Cd(II), while Sp adsorbed the smallest amount of Cd(II). Regardless of the type of adsorbent, the pH value of the solution after the adsorption of Cd(II) decreased to a different extent.

### 3.2. FTIR Spectra of Four Adsorbents Adsorbing Cd(II) Affected by Initial Cd(II) Concentration

**Figure 2:**
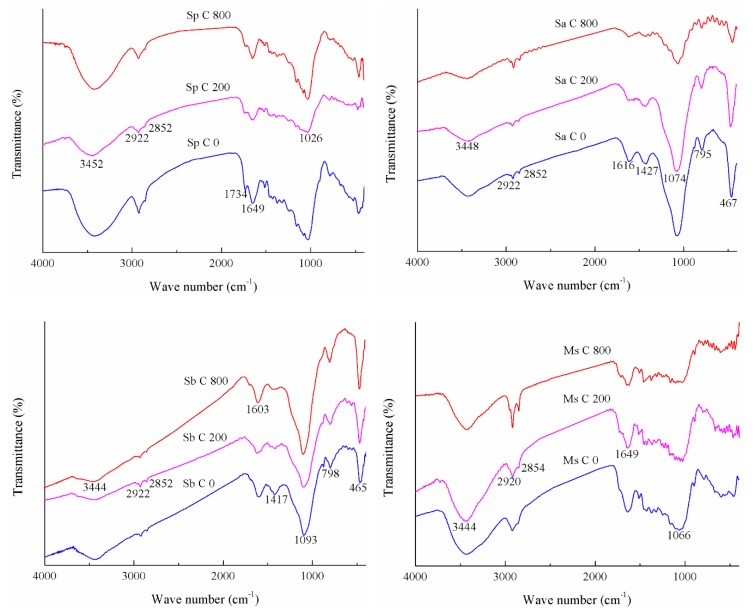
FTIR spectra of Cd(II) adsorbed by the four biosorbents under different initial Cd(II) concentrations. Note: the specific experiment conditions are as follows: the initial Cd(II) concentrations are set to 0, 200 and 800 mg/L, respectively, the ionic strength (NaCl) is 0.2 mol/L, and the initial pH value is not adjusted. The adsorption of Cd(II) on the rice straw powder (Sp), rice straw ash (Sa), rice straw biochar (Sb), and modified rice straw (Ms) at initial Cd(II) concentrations (C) 0, 200, and 800 mg/L are represented by Sp C 0, 200, 800, Sa C 0, 200, 800, Sb C 0, 200, 800, and Ms C 0, 200, 800, respectively.

As elucidated in Table 3 and Figure 2, with the increase in initial Cd(II) concentration from 0, 200, to 800 mg/L, the adsorption amount of Cd(II) by the four adsorbents also showed a gradual increase. The adsorption amount of Cd(II) by the four adsorbents was in the following order: Sa > Ms···Sb > Sp, which was slightly different from the order of their SSA.

Differences in oxygen-containing functional groups in the four adsorbents under different modification methods were presented in the pH section and will not be described again here. As the initial Cd(II) concentration and the amount of adsorption increased, the intensities of the absorption peaks at 3444~3452 and 1603~1649 cm^−1^ of the four adsorbents gradually decreased after adsorption. After the adsorption of Sa, Sb and Ms were completed, no absorption peak at 1734 cm^−1^ was observed. However, the intensity of this peak from Sp gradually decreased. The intensities of the absorption peaks at 1417~1427, 795~798 and 465~467 cm^−1^ of Sb decreased gradually and, when the initial Cd(II) concentration was 800 mg/L, a part of the absorption peak disappeared.

### 3.3. FTIR Spectra of Four Adsorbents Adsorbing Cd(II) Affected by Ionic Strength

**Figure 3:**
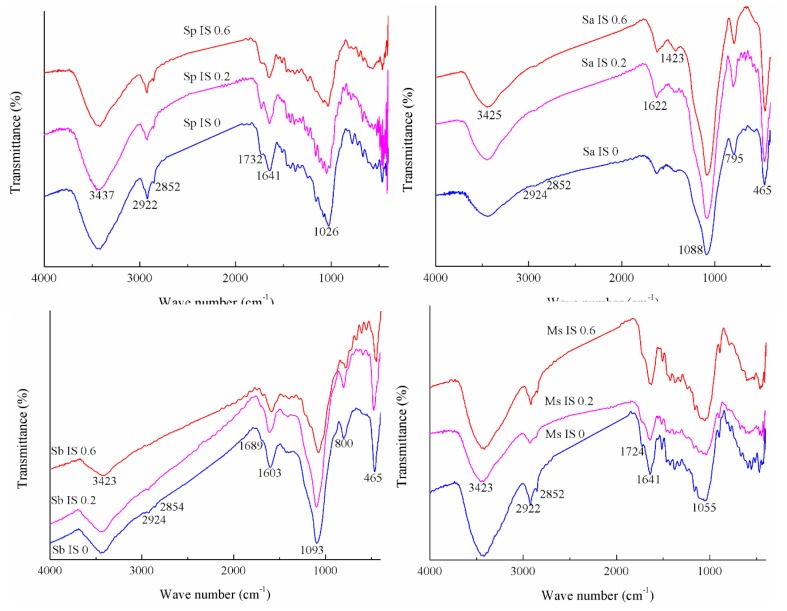
FTIR spectra of Cd(II) adsorbed by the four biosorbents under different ionic strengths. Note: the specific experiment conditions are as follows: the ionic strengths are set to 0, 0.2 and 0.6 mol/L, the initial Cd(II) concentration is 400 mg/L, and the initial pH value is not adjusted. The adsorption of Cd(II) on the rice straw powder (Sp), rice straw ash (Sa), rice straw biochar (Sb), and modified rice straw (Ms) at ionic strengths (IS) 0, 0.2, and 0.6 mol/L were represented by Sp IS 0, 0.2, 0.6, Sa IS 0, 0.2, 0.6, Sb IS 0, 0.2, 0.6, and Ms IS 0, 0.2, 0.6, respectively.

The effect of ionic strength on Cd(II) adsorption by the four adsorbents was investigated and illustrated in Table 4 and Figure 3. With the increase in ionic strength from 0, 0.2, to 0.6 mg/L, the adsorption amount of Cd(II) by Sa and Ms showed a gradual decrease, the same as the conclusion from El-Sayed et al. [34], but the adsorption amount of Cd(II) by Sp and Sb decreased greatly first and then increased slightly. Regardless of pH, initial Cd(II) concentration or ionic strength, Sa had an obvious advantage in the adsorption of Cd(II) over Sp, Sb, and Ms.

When the ionic strength was 0 mg/L, the amount of Cd(II) adsorbed by each adsorbent was the greatest, and the vibrational strength of the surface hydroxyl groups was the smallest. The intensity of the absorption peak at 1689~1732 cm^−1^ of Sp had the most advantage and, in this range, Ms and Sb had weaker intensity, but Sa had no peak at this position. The intensity of the absorption peak at 1603~1641 cm^−1^ of Ms was significantly higher than that of the other three adsorbents. This had similar laws under three influencing factors.

## 4. Discussion

Compared with three modified rice straws, the functional groups of raw rice straw were more complex and might mask the –O–CH_3_ group from lignin and Si–O–Si symmetric stretching. Incineration could remove large amounts of cellulose and hemicellulose, making the C=O group of a carboxylic acid or its ester of rice straw absent [14,21]. A portion of silica was removed after modification with citric acid, making Si–O–Si symmetric stretching disappear [16].

The pH value is considered as an important factor in Cd(II) sorption from aqueous solutions, as it affected both Cd(II) speciation and the protonation/deprotonation of functional groups on the adsorbents [1,8]. At lower pH values (2 and 4), the surface of the adsorbents was surrounded by H^+^, H_3_O^+^ ions occupying the sites [35], which prevented Cd(II) from approaching the binding sites on the adsorbents [12]. There existed electrostatic repulsion between the protonated functional groups and the positively charged Cd(II), which could impede the reaction with Cd(II) [36]. When the pH increased, the quantity of H^+^ in the solution decreased and the adsorption competition reduced correspondingly. The more negatively charged on the surface, the better the adsorption of Cd(II) [37]. Another aspect that should be considered is metal speciation in solution, which was also pH-dependent. An increase in the pH value of a solution resulted in the further hydrolysis of Cd(II) [6]. At a pH value of 9, several low-soluble hydroxyl complexes might be formed, such as Cd(OH)_2_ and Cd(OH)_3_^−^, which precipitated [22,38]. At this pH, possible formation of metal silicate precipitates such as CdSiO_3_ or Cd_2_SiO_4_ occurred [26]. It could be seen that the pH could strongly influence the adsorption performance of the four adsorbents and Cd(II) system, similar to Li et al. [6]’s conclusion. A decrease in pH value after adsorption in the adsorbents occurred since complexation with oxygen-containing functional groups was accompanied by the release of H^+^ to the solution [27]. The ion exchange mechanism could usually occur in the carboxylic and hydroxylic sites, which could be represented by the following reactions [39]:

2(≡XOH) + Cd^2+^ → (≡XO)_2_Cd + 2H^+^

2(≡XCOOH) + Cd^2+^ → (≡XCOO)_2_Cd + 2H^+^

Hence, the adsorption was predominantly due to an ion exchange mechanism. Additionally, since the adsorption greatly depended on the pH of the solution, the possible mechanism for Cd(II) adsorption by the adsorbents involved the coordination between Cd(II) and adsorbent functional groups [20]. Another mechanism might be the physical adsorption of Cd(II) depending on the characteristics of adsorbent, such as porosity and SSA [40]. The basis for the judgment was that straw ash had the largest SSA and pore volume, and the adsorbed amount of Cd(II) was also the greatest, but rice straw was the opposite. 

Changes in the chemical structure of the adsorbents are of vital importance in understanding the adsorption process. The FTIR technique is an important tool to identify the characteristic functional groups, which are instrumental in the adsorption of Cd(II) [17]. In the four adsorbents, the hydroxylic sites were the predominant acidic sites and were associated with the hydroxyls of the cellulose and hydroxylic and phenolic groups of the lignin [39], which might help to remove Cd(II) in aqueous solution [20]. The pyrolysis of the rice straw in an oxygen-limited environment and incineration in an aerobic environment to produce rice straw biochar and ash, which could somewhat reduce their cellulose and lignin contents, made the intensities of peaks at 3419 and 2922 cm^−1^ decrease. At this time, their surfaces were dominated by the silica functional groups of Si–O (1080~1097 cm^−1^) and Si–O–Si (796~804 cm^−1^) [31]. The band at 1720 cm^−1^ was absent in straw ash as a result of the removal of hemicelluloses from the rice straw [41]. In addition, it was also possible that the surface carboxylic acid and Cd(II) were firmly bonded, making the peak at 1720 cm^−1^ disappear. In contrast, some silica was removed after CA modification of rice straw owing to the depolymerization and decomposition of cellulose, hemicelluloses, and lignin [21]. The CA was first dehydrated by heating and converted to a reactive hydride that reacted with hydroxyls in the cellulose and lignin to form an ester [8]. Therefore, the CA molecules bound to the rice straw through the esterification reaction and, in this way, more than two carboxylic sites were added to each bound CA molecule [39], which made the carboxylic acid content in this modified rice straw higher than other adsorbents. After the silica was removed, the groups of –OH acids were more exposed on the modified straw surfaces, possibly resulting in a stronger interaction with water molecules [16]. Coordination with delocalized *π*-electrons (e.g., C=C, C=O) might have also played an important role in Cd(II) adsorption [33]. In the spectra of rice straw biochar, rice straw ash, and modified rice straw after the adsorption of Cd(II), an increase in the asymmetric stretching vibration of C=O in the ionic carboxylic groups (−COO^−^) at 1605~1637 cm^−1^ was observed, indicating more carboxyl bindings than the crude rice straw without any modification [42]. 

For the four adsorbents, the surface hydroxyl group, –O–CH_3_ group from lignin, and Si–O group were the most important oxygen-containing functional groups. Compared with rice straw, the rice straw biochar, ash, and modified rice straw were more likely to act as *π* donors in the Cd(II) sorption process [1]. Cd(II)–*π* interactions usually occurred between Cd(II) and electron-rich double bonds or triple bonds [2], and this surface complexation was a major adsorption mechanism in the Cd(II) sorption process. After Cd(II) sorption, the peaks at 1605~1637 cm^−1^ (assigned to C=C aromatic stretching of lignin) of the four adsorbents weakened gradually, suggesting that the aromatic C=C was involved in the Cd(II) sorption process [1]. As pH value of the solution increased, the input of OH^−^ from the external source increased, which promoted the association of OH^−^ with Cd(II) and, in addition, neutralized the carboxyl groups on the surface of the adsorbents. At a pH value of 2, the cellulose, hemicellulose, and lignin of the four adsorbents might be loosened and converted to glucose [43], which would contribute to the greatest intensity of the peak at 1020~1097 cm^−1^ among the three pH values. Many more bond stretches appearing around 1419~1423 cm^−1^ after Cd(II) sorption on them was equally an indication that Cd(II)–*π*–electron interaction took place during sorption [33]. The introduction of –O–CH_3_ could promote the adsorption of Cd(II) on the carbon surface [10].

An increase in the initial Cd(II) concentration helped overcome the all mass transfer resistance of Cd(II) between the liquid and solid phases [30]. The increase in adsorption capacity was due to the increase in the driving force of Cd(II) to the adsorption sites [37]. For rice straw ash, the higher the SSA (192.38 m^2^/g) was, the greater its adsorption capacity for Cd(II) [44]. In contrast, the rice straw with the least SSA (1.83 m^2^/g) had the least adsorptive capacity. However, the order of the adsorption of Cd(II) by the four adsorbents was different from the order of SSA, which indicated that in addition to electrostatic attraction, there were other mechanisms controlling the adsorption. The functional groups and Cd(II) from the adsorbents derived from rice straw might have undergone a chemical reaction [39]. The increased Cd(II) concentration occupied more surface hydroxyl groups so that the intensity of O–H vibration significantly reduced after interaction with Cd(II) [26]. A pronounced decrease was visible in the C=C band of the aromatic rings (1603~1649 cm^−1^), which indicated that the polarity of the new molecule from the four adsorbents after adsorbing Cd(II) had changed [39]. The interaction between the four adsorbents and Cd(II) generated a change in the dipole moment in the double bonds, which might indicate a *π*–cation interaction between the lignin aromatic rings and Cd(II) [39]. Furthermore, for rice straw, the complexation of Cd(II) with the carboxyl group was one of the main mechanisms for its adsorption of Cd(II) [45]. The absences of COO^−^ in the rice straw biochar, ash, and modified rice straw indicated that there was an interaction between the carboxylic sites of the three adsorbents mentioned above and the Cd(II) or other cations in the solution. In other words, Cd(II) and other cations were adsorbing on the less carboxylic sites [39], making the peak disappear. Asuquo et al. [46] also pointed out that the decrease in the wavenumber of the peak at 1734 cm^−1^ that was a characteristic for the C=O group from the carboxylic acid and its disappearance could be used as direct evidence for the interaction between Cd(II) and these adsorbents during adsorption. The –O–CH_3_ group of lignin, and Si–O–Si or Si–O groups from the rice straw biochar participated in the association with Cd(II). When the initial Cd(II) concentration reached 800 mg/L, the disappearance of the absorption peaks at 1417 and 798 cm^−1^ after the adsorption of Cd(II) by rice straw biochar might be related to the formation of CdSiO_3_ or Cd_2_SiO_4_ [26].

The ionic strength of the solution may be an important factor influencing aqueous phase equilibrium between adsorbed species and adsorbent [34]. The effect of ionic strength on Cd(II) adsorption on the four adsorbents is achieved by varying the concentrations of additive NaCl from 0, 0.2, to 0.6 mol/L. The presence of Na(I) significantly suppressed the adsorption of Cd(II) onto the rice straw ash and modified rice straw due to the competitive adsorption occurred [30]. For rice straw powder and biochar, compared with an ionic strength of 0 mol/L, the inhibition of Cd(II) adsorption by the ionic strength of 0.2 mol/L could also be explained by the competition of the limited binding sites on the surface of adsorbents [47]. However, a higher ionic strength (0.6 mol/L) could not completely make the active sites of rice straw powder and biochar occupied, and an electrical diffused double layer occurred by Na(I) caused a repulsion of Cd(II) in the solution [48], making the adsorption capacity weaken a little. The carboxyl group of the rice straw ash was more easily associated with Cd(II) and its absorption peak disappeared. The absorption intense for C=O groups was increased in the modified rice straw, since the acid hydrolysis reaction might cause partial lignin structure to release [49]. It might be generated by the cleavage of ether bonds within the lignin [49].

When there was no ionic strength interference or no competitive adsorption, more function groups were available for Cd(II) uptake, and the degree of association between Cd(II) and the hydroxyl group on the surface of the adsorbent was the greatest, so that the vibration intensity of the absorption peak at 3425~3437 cm^−1^ was the smallest. Similar results were reported by Chen et al. [50]. As the ionic strength increased, the adsorption amount of Cd(II) decreased to varying degrees, and the vibration intensity of the absorption peak also decreased. The reason why it did not increase gradually was that the combination of adding Na(I) and surface hydroxyl groups could also reduce the peak intensity to some extent. The ionic strength-dependent adsorption indicated that cation exchange partly contributed to the adsorption [51,52].

The appearance of the absorption peak at 1020~1097 cm^−1^ resulted from the overlap of C–O or the C–O–C stretching of the –O–CH_3_ group from lignin and Si–O stretching vibration, so it could provide more useful information when comparing the surface characteristics among different adsorbents, but it was not sensitive to the adsorption of Cd(II) by the adsorbent under different pH values and ionic strengths. In addition, if the amounts of oxygenated groups in four adsorbents could be quantified via Boehm titration and XPS tests for the possible transformation during the surficial sorption, more valuable information would be provided. Some important models and control experiments were also worth exploring, such as cadmium acetate, cadmium benzoate, cadmium phenol, cadmium citrate, etc., which would enrich the research content.

## 5. Conclusions

Compared with Sp, Sb and Sa contained fewer hydroxyl and alkyl groups, but more Si–O groups. A portion of silica from Ms was removed by citric acid, and thus the carboxylic content of Ms was increased, and more –OH groups of Ms were exposed. Compared with Sp, Sb, Sa, and Ms were more likely to act as *π* donors in the Cd(II) sorption process and had exhibited carboxyl binding. The bands of C=C, –O–CH_3_, and the O–H, carboxyl, Si–O–Si or Si–O groups were involved in the Cd(II) sorption process. The adsorption amount of Cd(II) by the four adsorbents increased with the increase in the pH value of the solution and the initial Cd(II) concentration. Ion exchange, surface complexation, and precipitation were the major adsorption mechanisms in the effect of the initial pH value, while the latter electrostatic attraction played a leading role in the effect of the initial Cd(II) concentration. With no interference by ionic strength, all the adsorbents had the greatest adsorption amount of Cd(II), and the intensity of O–H vibration was also the weakest; ion exchange was the most important mechanism in this process. Regardless of the influencing factors, Sa, with the greatest specific surface area, had an absolute advantage in the adsorption capacity of Cd(II) over Sp, Sb, and Ms. In particular in optimum environmental conditions where the initial Cd(II) concentration was 800 mg/L, the pH value was 9, and there was no other ion interference, Sa had the ability to remove Cd(II) pollution in aqueous systems more strongly.

## Figures and Tables

**Table 1:** Positions and Spectral Assignments of Cd(II) Adsorbed by the Four Biosorbents.

Positions (cm^−1^)	Spectral Assignments	References
3404~3452	Free and intermolecular bonded hydroxyl groups	Vázquez et al. [23] Song et al. [3]
2920~2924	C–H stretching vibration of –CH_2_	Vázquez et al. [23] Lin et al. [9] Saeed et al. [24]
2852~2854	C–H stretching vibration of –CH_3_	Pap et al. [30] Park et al. [21]
1689~1734	C=O group of a carboxylic acid or its ester	Vázquez et al. [23]
1603~1649	C=C stretching of aromatic or benzene rings in lignin	Vázquez et al. [23] Lin et al. [9]
1512	N–H bending of secondary aromatic amines	Saeed et al. [24] Park et al. [21]
1417~1427	C–H deformation of –O–CH_3_ group from lignin	Pap et al. [30] Vázquez et al. [23]
1020~1097	C–O or C–O–C stretching of –O–CH_3_ group from lignin or Si–O stretching vibration	Zhu et al. [8] Rocha et al. [16] Zheng et al. [18] Nakbanpote et al. [31]
795~804	Si–O–Si symmetric stretching	Qian and Chen [32]
451~467	Si–O bending vibration	Rocha et al. [16] Qian and Chen [32]

**Table 2:** Adsorption Capacity of Cd(II) on the Four Adsorbents from Rice Straw under Different Initial Cd(II) Concentrations and the Relative Intensities of the Absorption Peaks.

Treatmets	Initial pH Value	Final pH Value	*q_e_* (mg/g)	Peak Positions (cm^−1^) and Their Relative Intensities (%)								
				3404	2920	2852	1720	1637	–	1020	–	459
Sp pH 2	2	0.61	2.2cC	41.31aB	1.40aB	0.02cD	0.57aC	2.24aD	–	44.62aC	–	9.83cA
Sp pH 4	4	2.24	4.7bD	39.25bB	1.30bB	0.19aA	0.34bC	2.12bD	–	38.13cC	–	18.67aA
Sp pH 9	9	4.63	11.3aD	37.88cB	1.25bB	0.08bC	0.11cC	2.00cD	–	43.79bC	–	14.89bA
				3419	2922	2852	–	1620	1421	1080	804	467
Sa pH 2	2	0.66	3.53cA	26.71aC	0.40bD	0.13aB	–	3.67aC	0.00b	61.35aA	2.07a	5.66cC
Sa pH 4	4	3.54	16.3bA	25.87bC	0.64aC	0.10bC	–	3.55bC	0.00bB	61.04aA	2.00ab	6.80bC
Sa pH 9	9	6.82	60.0aA	22.77cC	0.44bD	0.13aB	–	3.14cC	2.67aB	60.13bA	1.98b	8.75aB
				3419	2922	2852	1693	1605	1419	1097	796	463
Sb pH 2	2	1.29	3.66cA	23.31aD	0.56bC	0.06cC	0.65aB	4.25aB	0.00c	59.79aB	4.31a	7.06cB
Sb pH 4	4	2.71	5.59bC	21.77bD	0.56bD	0.15bB	0.56bB	4.06bB	1.77bA	59.34bB	4.03b	7.76bB
Sb pH 9	9	4.86	12.7aC	19.22cD	0.79aC	0.24aA	0.14cB	3.93cB	3.73aA	59.25bB	3.53c	7.97aC
				3415	2920	2852	1726	1633	1423	1051	–	451
Ms pH 2	2	0.62	2.43cB	56.21aA	3.04bA	0.19aA	0.89aA	4.97aA	0.00b	34.21aD	–	0.00cD
Ms pH 4	4	2.12	9.39bB	54.49bA	2.87cA	0.09bC	0.77bA	4.57bA	0.00bB	33.91bD	–	2.83aD
Ms pH 9	9	5.31	31.9aB	53.27cA	3.88aA	0.16aB	0.40cA	4.41cA	2.06aC	33.50cD	–	2.00bD

Note: *q_e_*(mg/g) indicates the amount of Cd(II) adsorption at equilibrium. The adsorption of Cd(II) on the rice straw powder (Sp), rice straw ash (Sa), rice straw biochar (Sb), and modified rice straw (Ms) at pH values 2, 4, and 9 are represented by Sp pH 2, 4, 9, Sa pH 2, 4, 9, Sb pH 2, 4, 9, and Ms pH 2, 4, 9, respectively. Different lower-case letters indicate a significant difference among different pH values at the same adsorbent, and different capital letters indicate a significant difference between the different types of adsorbent at the same pH value (*p* < 0.05).

**Table 3:** Adsorption Capacity of Cd(II) on the Four Adsorbents from Rice Straw under Different Initial Cd(II) Concentrations and the Relative Intensities of the Absorption Peaks.

Treatments	*C_0_* (mg/L)	*q_e_* (mg/g)	Peak Positions (cm^−1^) and Their Relative Intensities (%)								
			3452	2922	2852	1734	1649	–	1026	–	–
Sp C 0	0	0c	47.58aB	1.70bB	0.05bC	0.88a	4.36aC	–	45.44cC	–	–
Sp C 200	200	3.11bC	46.29bB	2.16aB	0.16aC	0.55b	4.23bC	–	46.61bC	–	–
Sp C 800	800	5.83aC	46.24bB	1.34cB	0.05bD	0.40c	3.77cB	–	48.20aC	–	–
			3448	2922	2852	–	1616	1427	1074	795	467
Sa C 0	0	0c	21.93aD	0.59bD	0.15bB	–	4.01aD	2.49a	63.20cA	2.22a	5.41a
Sa C 200	200	44.5bA	20.34bD	0.59bD	0.12bD	–	2.28bD	2.15b	67.05bA	2.16b	5.30b
Sa C 800	800	56.1aA	20.12cD	0.96aC	0.86aB	–	1.93cC	1.92c	70.11aB	0.00c	4.10c
			3444	2922	2852	–	1603	1417	1093	798	465
Sb C 0	0	0c	23.80aC	0.69cC	0.13bB	–	6.99aB	3.26a	53.63cB	4.55a	6.94a
Sb C 200	200	5.32bB	22.57bC	1.02aC	0.28aA	–	6.68bB	2.01b	56.72bB	4.16b	6.58b
Sb C 800	800	5.94aC	21.32cC	0.85bD	0.15bC	–	1.36cD	0.00c	71.28aA	0.00c	5.03c
			3444	2920	2854	–	1649	–	1066	–	–
Ms C 0	0	0c	57.46aA	2.85bA	0.20cA	–	9.00aA	–	30.49bD	–	–
Ms C 200	200	5.33bB	57.21aA	2.53cA	0.25bB	–	8.90bA	–	31.11aD	–	–
Ms C 800	800	7.39aB	52.30bA	7.57aA	1.62aA	–	8.67cA	–	29.85cD	–	–

Note: *C_0_* (mg/L) and *q_e_*(mg/g) indicate the initial concentration of Cd(II) and the amount of Cd(II) adsorption at equilibrium, respectively. The adsorption of Cd(II) on the rice straw powder (Sp), rice straw ash (Sa), rice straw biochar (Sb), and modified rice straw (Ms) at initial Cd(II) concentrations (C) 0, 200, and 800 mg/L are represented by Sp C 0, 200, 800, Sa C 0, 200, 800, Sb C 0, 200, 800, and Ms C 0, 200, 800, respectively. Different lower-case letters indicate a significant difference among different initial Cd(II) concentrations at the same adsorbent, and different capital letters indicate a significant difference between the different types of adsorbent at the same initial Cd(II) concentration (*p* < 0.05).

**Table 4:** Adsorption Capacity of Cd(II) on the Four Adsorbents from Rice Straw under Different Ionic Strengths and the Relative Intensities of the Absorption Peaks.

Treatments	Ionic Strength (mg/L)	*q_e_* (mg/g)	Peak Positions (cm^−1^) and Their Relative Intensities (%)								
			3437	2922	2852	1732	1641	–	1026	–	–
Sp IS 0	0	12.6aD	56.17cB	2.11bB	0.22aB	1.23aA	3.29cC	–	36.98aC	–	–
Sp IS 0.2	0.2	4.5cC	56.48bB	1.86cA	0.10cA	1.16bA	3.82aC	–	36.58bC	–	–
Sp IS 0.6	0.6	4.8bD	58.89aA	2.24aB	0.19bB	1.06cA	3.61bC	–	34.02cC	–	–
			3425	2924	2852	–	1622	1423	1088	795	465
Sa IS 0	0	48.8aA	23.35cD	0.19aD	0.06aD	–	2.93cD	0.67b	61.53bA	2.65c	8.63a
Sa IS 0.2	0.2	25.6bA	25.88aC	0.07bC	0.02bB	–	3.64bD	0.63c	59.97cA	2.82a	6.96b
Sa IS 0.6	0.6	15.4cA	24.00bD	0.04cD	0.03bD	–	3.75aB	0.76a	62.51aB	2.74b	6.16c
			3423	2924	2854	1689	1603	–	1093	800	465
Sb IS 0	0	14.5aB	24.22cC	0.43aC	0.09bC	0.14aB	5.10bB	–	56.67cB	3.28a	8.06a
Sb IS 0.2	0.2	4.7cC	25.73bD	0.09cC	0.11aA	0.14aB	5.23aB	–	59.45bB	3.19b	6.06b
Sb IS 0.6	0.6	5.4bB	31.62aC	0.11bC	0.11aC	0.00bB	1.24cD	–	64.48aA	0.00c	2.43c
			3423	2922	2852	1724	1641	–	1055	–	–
Ms IS 0	0	13.7aC	56.98bA	2.53aA	0.38aA	0.13aB	7.33bA	–	32.66aD	–	–
Ms IS 0.2	0.2	7.2bB	59.06aA	1.50bB	0.11cA	0.11bC	7.29bA	–	31.94bD	–	–
Ms IS 0.6	0.6	5.1cC	57.09bB	2.56aA	0.22bA	0.00cB	8.13aA	–	31.99bD	–	–

Note: *q_e_*(mg/g) indicates the amount of Cd(II) adsorption at equilibrium. The adsorption of Cd(II) on the rice straw powder (Sp), rice straw ash (Sa), rice straw biochar (Sb), and modified rice straw (Ms) at ionic strengths (IS) 0, 0.2, and 0.6 mol/L are represented by Sp IS 0, 0.2, 0.6, Sa IS 0, 0.2, 0.6, Sb IS 0, 0.2, 0.6, and Ms IS 0, 0.2, 0.6, respectively. Different lower-case letters indicate a significant difference among different ionic strengths at the same adsorbent, and different capital letters indicate a significant difference between the different types of adsorbent at the same ionic strength (*p* < 0.05).

## References

[1] Cui X.Q., Fang S.Y., Yao Y.Q., Li T.Q., Ni Q.J., Yang X.E., He Z.L. (2016). Potential mechanisms of cadmium removal from aqueous solution by *Canna indica* derived biochar. Sci. Total Environ..

[2] Peng H.B., Gao P., Chu G., Pan B., Peng J.H., Xing B.S. (2017). Enhanced adsorption of Cu(II) and Cd(II) by phosphoric acid-modified biochars. Environ. Pollut..

[3] Song M., Wei Y.X., Cai S.P., Yu L., Zhong Z.P., Jin B.S. (2018). Study on adsorption properties and mechanism of Pb^2+^ with different carbon based adsorbents. Sci. Total Environ..

[4] Sharma R., Singh B. (2013). Removal of Ni(II) ions from aqueous solutions using modified rice straw in a fixed bed column. Bioresource Technol..

[5] Wu W.Y., Yao G.C., Zhang X.W., Chen Z.M., Zhang X.F., Chen D.Y. (2015). Adsorption behavior of modified rice straw to thorium. Nuclear Techniques.

[6] Li W.C., Law F.Y., Chan Y.H.M. (2017). Biosorption studies on copper (II) and cadmium (II) using pretreated rice straw and rice husk. Environ. Sci. Pollut. Res..

[7] Binod P., Sindhu R., Singhania R., Vikram S., Devi L., Nagalakshmi S., Kurien N., Sukumaran R., Pandey A. (2010). Bioethanol production from rice straw: An overview. Bioresour. Technol..

[8] Zhu B., Fan T.X., Zhang D. (2008). Adsorption of copper ions from aqueous solution by citric acid modified soybean straw. J. Hazard Mater..

[9] Lin C., Luo W.J., Luo T.T., Zhou Q., Li H.F., Jing L.R. (2018). A study on adsorption of Cr (VI) by modified rice straw: Characteristics, performances and mechanism. J. Clean Prod..

[10] Yang X.D., Wan Y.S., Zheng Y.L., He F., Yue Z.B., Huang J., Wang H.L., Ok Y.S., Jiang Y.S., Gao B. (2019). Surface functional groups of carbon-based adsorbents and their roles in the removal of heavy metals from aqueous solutions: A critical review. Chem. Eng. J..

[11] Li H.B., Dong X.L., da Silva E.B., de Oliveira L.M., Chen Y.S., Ma L.Q. (2017). Mechanisms of metal sorption by biochars: Biochar characteristics and modifications. Chemosphere.

[12] Wong K.K., Lee C.K., Low K.S., Haron M.J. (2003). Removal of Cu and Pb by tartaric acid modified rice husk from aqueous solutions. Chemosphere.

[13] Rungrodnimitchai S. (2014). Rapid preparation of biosorbents with high ion exchange capacity from rice straw and bagasse for removal of heavy metals. Sci. World J..

[14] Ahmaruzzaman M., Gupta V.K. (2011). Rice husk and its ash as low-cost adsorbents in water and wastewater treatment. Ind. Eng. Chem. Res..

[15] Nnaji C.C., Ebeagwu C.J., Ugwu E.I. (2017). Physicochemical conditions for adsorption of lead from water by rice husk ash. BioRes.

[16] Rocha C.G., Zaia D.A.M., Alfaya R.V.D.S., Alfaya A.A.D.S.A. (2009). Use of rice straw as biosorbent for removal of Cu(II), Zn(II), Cd(II) and Hg(II) ions in industrial effluents. J. Hazard Mater..

[17] Ye H.P., Zhu Q., Du D.Y. (2010). Adsorptive removal of Cd(II) from aqueous solution using natural and modified rice husk. Bioresource Technol..

[18] Zheng L.C., Dang Z., Yi X.Y., Zhang H. (2010). Equilibrium and kinetic studies of adsorption of Cd(II) from aqueous solution using modified corn stalk. J. Hazard Mater..

[19] Pehlivan E., Altun T., Parlayici Ş. (2012). Modified barley straw as a potential biosorbent for removal of copper ions from aqueous solution. Food Chem.

[20] Cheng Q.M., Huang Q., Khan S., Liu Y.J., Liao Z.N., Li G., Ok Y.S. (2016). Adsorption of Cd by peanut husks and peanut husk biochar from aqueous solutions. Ecol. Eng..

[21] Park J.H., Wang J.J., Kim S.H., Cho J.S., Kang S.W., Delaune R.D., Han K.J., Seo D.C. (2017). Recycling of rice straw through pyrolysis and its adsorption behaviors for Cu and Zn ions in aqueous solution. Colloid Surface A.

[22] Iqbal M., Saeed A., Zafar S.I. (2009). FTIR spectrophotometry, kinetics and adsorption isotherms modeling, ion exchange, and EDX analysis for understanding the mechanism of Cd^2+^ and Pb^2+^ removal by mango peel waste. J. Hazard Mater..

[23] Vázquez G., Freire M.S., González-Alvarez J., Antorrena G. (2009). Equilibrium and kinetic modeling of the adsorption of Cd^2+^ ions onto chestnut shell. Desalination.

[24] Saeed A., Iqbal M., Höll W.H. (2009). Kinetics, equilibrium and mechanism of Cd^2+^ removal from aqueous solution by mungbean husk. J. Hazard. Mater..

[25] Li B., Yang L., Wang C.Q., Zhang Q.P., Liu Q.C., Li Y.D., Xiao R. (2017). Adsorption of Cd(II) from aqueous solutions by rape straw biochar derived from different modification processes. Chemosphere.

[26] Gao L.Y., Deng J.H., Huang G.F., Li K., Cai K.Z., Liu Y., Huang F. (2019). Relative distribution of Cd^2+^ adsorption mechanisms on biochars derived from rice straw and sewage sludge. Bioresource Technol..

[27] Cao X., Ma L., Gao B., Harris W. (2009). Dairy-manure derived biochar effectively sorbs lead and atrazine. Environ. Sci. Technol..

[28] Bashir S., Zhu J., Fu Q.L., Hu H.Q. (2018). Comparing the adsorption mechanism of Cd by rice straw pristine and KOH-modified biochar. Environ. Sci. Pollut. R..

[29] Che Z.A.A., Naimah I., Fahmi M.R., Salsuwanda S. (2014). Removal of Cu(II) from industrial effluents by citric acid modified rice straw. Bioremediation Sci. Technol. Res..

[30] Pap S., Radonić J., Trifunović S., Adamović D., Mihajlović I., Miloradov M.V., Sekulić M.T. (2016). Evaluation of the adsorption potential of eco-friendly activated carbon prepared from cherry kernels for the removal of Pb^2+^, Cd^2+^ and Ni^2+^ from aqueous wastes. J. Environ. Manage..

[31] Nakbanpote W., Goodman B.A., Thiravetyan P. (2007). Copper adsorption on rice husk derived materials studied by EPR and FTIR. Colloid Surface A.

[32] Qian L., Chen B. (2013). Dual role of biochars as adsorbents for aluminum: the effect of oxygen-containing organic components and scattering of silicate particles. Environ. Sci. Technol..

[33] Zama E.F., Zhu Y.G., Reid B.J., Sun G.X. (2017). The role of biochar properties in influencing the sorption and desorption of Pb(II), Cd(II) and As(III) in aqueous solution. J. Clean Prod..

[34] El-Sayed G.O., Dessouki H.A., Ibrahim S.S. (2010). Biosorption of Ni(II) and Cd(II) ions from aqueous solutions onto rice straw. Chem. Sci. J..

[35] Zheng L.C., Zhu C.F., Dang Z., Zhang H., Yi X.Y., Liu C.Q. (2012). Preparation of cellulose derived from corn stalk and its application for cadmium ion adsorption from aqueous solution. Carbohyd. Polym..

[36] Ding Y., Jing D.B., Gong H.L., Zhou L.B., Yang X.S. (2012). Biosorption of aquatic cadmium(II) by unmodified rice straw. Bioresource Technol..

[37] Song T., Liang J.S., Bai X., Li Y., Wei Y.N., Huang S.Q., Dong L.Y., Qu J.J., Jin Y. (2017). Biosorption of cadmium ions from aqueous solution by modified *Auricularia Auricular* matrix waste. J. Mol. Liq..

[38] Mohan D., Pittman C.U., Bricka M., Smith F., Yancey B., Mohammad J., Steele P.H., Alexandre-Franco M.F., Gómez-Serrano V., Gong H. (2007). Sorption of arsenic, cadmium, and lead by chars produced from fast pyrolysis of wood and bark during bio-oil production. J. Colloid Interface Sci..

[39] Leyva-Ramos R., Landin-Rodriguez L.E., Leyva-Ramos S., Medellin-Castillo N.A. (2012). Modification of corncob with citric acid to enhance its capacity for adsorbing cadmium(II) from water solution. Chem. Eng. J..

[40] Liu Z., Zhang F.S. (2011). Removal of copper (II) and phenol from aqueous solution using porous carbons derived from hydrothermal chars. Desalination.

[41] Hsu T.C., Guo G.L., Chen W.H., Hwang W.S. (2010). Effect of dilute acid pretreatment of rice straw on structural properties and enzymatic hydrolysis. Bioresource Technol..

[42] Liang S., Guo X.Y., Tian Q.H. (2011). Adsorption of Pb^2+^ and Zn^2+^ from aqueous solutions by sulfured orange peel. Desalination.

[43] Nasr M., Mahmoud A.E.D., Fawzy M., Radwan A. (2017). Artificial intelligence modeling of cadmium(II) biosorption using rice straw. Appl. Water Sci..

[44] Wu M.J., Liu H.Y., Yang C.P. (2019). Effects of pretreatment methods of wheat straw on adsorption of Cd(II) from waterlogged paddy soil. Int. J. Environ. Res. Public Health.

[45] Chen T., Zhou Z.Y., Han R., Meng R.H., Wang H.T., Lu W.J. (2015). Adsorption of cadmium by biochar derived from municipal sewage sludge: Impact factors and adsorption mechanism. Chemosphere.

[46] Asuquo E.D., Martin A.D., Nzerem P. (2018). Evaluation of Cd(II) ion removal from aqueous solution by a low-cost adsorbent prepared from white yam (*Dioscorea rotundata*) waste using batch sorption. ChemEngineering.

[47] Vasudevan P., Padmavathy V., Dhingra S.C. (2002). Biosorption of monovalent and divalent ions on baker’s yeast. Bioresource Technol..

[48] Salmani M.H., Zarei S., Ehrampoush M.H., Danaie S. (2013). Evaluations of pH and high ionic strength solution effect in cadmium removal by zinc oxide nanoparticles. J. Appl. Sci. Environ. Manage.

[49] Zhao J.S., Fu C.G., Yang Z.Y. (2008). Integrated process for isolation and complete utilization of rice straw components through sequential treatment. Chem. Eng. Comm..

[50] Chen Y.G., Ye W.M., Yang X.M., Deng F.Y., He Y. (2011). Effect of contact time, pH, and ionic strength on Cd(II) adsorption from aqueous solution onto bentonite from Gaomiaozi, China. Environ. Earth Sci..

[51] Baeyens B., Bradbury M.H. (1997). A mechanistic description of Ni and Zn sorption on Na-montmorillonite. Part I: Titration and sorption measurements. J. Contam. Hydrol..

[52] Wang X.K., Liu X.P. (2004). Effect of pH and concentration on the diffusion of radiostrontium in compacted bentonite—A capillary experimental study. Appl. Radiat. Isot..

